# Biological Activities of Four Adaptogenic Plant Extracts and Their Active Substances on a Rotifer Model

**DOI:** 10.1155/2018/3690683

**Published:** 2018-10-14

**Authors:** Lilla Mácsai, Zsolt L. Datki, Dezső Csupor, Attila Horváth, Zoltán P. Zomborszki

**Affiliations:** ^1^Department of Psychiatry, University of Szeged, Faculty of Medicine, Szeged 6720, Hungary; ^2^Department of Pharmacognosy, University of Szeged, Faculty of Pharmacy, Szeged 6720, Hungary

## Abstract

Rotifers have been widely used as well-characterized models of aging, since their multiorgan character makes them suitable as* in vivo* toxicological and lifespan models. Here we report the assessment of four adaptogenic plants and their extracts for the first time in this model. The effects on rotifer viability of extracts and characteristic active markers of* Panax ginseng*,* Withania somnifera*,* Leuzea carthamoides,* and* Rhodiola rosea* were tested* in vivo*. The crude extracts were nontoxic to* Philodina acuticornis* bdelloid rotifers; however, the pure substances of the plants influenced negatively the viability. Ginsenoside Rb1 and secondary metabolites of* Withania somnifera* exerted deleterious effect on the animals. The aglycone tyrosol and cinnamyl alcohol (from* Rhodiola rosea*) were more toxic than their glycosides salidroside and rosavin. Although the 20-OH-ecdysone and ajugasterone C (from* Leuzea carthamoides*) are chemically very similar, the latter was less toxic.

## 1. Introduction

*Panax ginseng *Meyer,* Withania somnifera *(L.) Dunal,* Leuzea carthamoides* Willd., and* Rhodiola rosea* L. have been widely used in the folk medicine for their adaptogenic properties, to maintain physical and mental health. Their health benefits have been exploited for centuries; thus extensive biological and chemical researches have been conducted to explore their active substances and further possible applications of these plants. In Europe* Withania frutescens* is native and may be a good alternative of* W. somnifera*, due to their similar chemical composition [1]. Despite the benefits of these plants in different animal models and human settings, the biological effects of* P. ginseng, W. frutescens, L. carthamoides*, and* R. rosea* have not yet been examined on rotifer model.

Rotifers (phylum Rotifera) are widely used research models in the fields of ecotoxicology and aging [2, 3]. The bdelloids, with their multiorgan characters and sensitivity for chemicals [4], together with the short lifespan and specific measurable phenotypic features and viability markers [5] are useful as* in vivo* toxicological and lifespan models. Due to their size, these animals have outstanding advantages in terms of culturing and are rather easy to work with [5, 6]. The reproduction of bdelloid rotifers is also with obligatory parthenogenesis (asexual) and males have been extinct for more than 30 million years [7]. There are a variety of phenotypic characteristics available to* in vivo* experimental settings. The rotifers' body size is in correlation with lifetime [8]. The mastax (pharynx) is part of the digestive system, with the function to shred the food by periodic opening and closing [9]. The functioning of the mastax is a quantitative indicator of viability [5, 10]. The aim of our work was to study the biological effect of the above-mentioned four adaptogenic plants on rotifers in order to reveal their toxic and pharmacologically perspective effects in this model.

## 2. Materials and Methods

### 2.1. Herbal Extracts and Compounds

Plant materials were purchased from local market (*P. ginseng*) or collected from culture (*W. frutescens, L. carthamoides, *and* R. rosea*) and identified by the authors. Extraction of plant materials was carried out with 50% of ethanol for* P. ginseng, W. frutescens,* and* L. carthamoides*. In the case of* R. rosea, *a 70% ethanol extract was used. 1.00 gram of plant material was extracted with 10.0 ml of extraction solvent for 10 minutes in ultrasonic bath at room temperature, and then the extracts were evaporated to dryness. Ginsenoside Rb1 was purchased from HWI Analytik Gmbh (Tübingen, Germany), withanolide A, withanolide B, and withaferin A were purchased from Phytolab (Vestenbergsgreuth, Germany), rosavin, salidrosid, tyrosol, and cinnamyl alcohol were purchased from Sigma-Aldrich (Düsseldorf, Germany), rhodiosin was purchased from Carbosynth (Compton-Burkshire, UK), and 20-OH-ecdysone and ajugasterone were isolated in our Department. The structure and purity of the isolated materials have been verified via NMR and MS analysis.

### 2.2. HPLC Analysis

Solutions of redissolved extracts (25 mg/mL) were filtered through 0.45 *μ*m PTFE syringe filters and characterized chemically by HPLC-DAD by quantifying some biologically active markers of the plants. HPLC analysis was carried out using a HPLC system comprising a Shimadzu LC-20AD pump, DGU-20A5R degasser, SIL-20ACH autosampler (tempered to 21°C), CTO-20AC column oven, and SPD-M20A photodiode array detector modules, connected with CBM-20A control module. Column temperature was set to 25°C. The solvent system for quantification of* W. frutescens, P. ginseng,* and* L. carthamoides* consisted of 0.1% H_3_PO_4_ (A) and acetonitrile (B). In the case of* W. frutescens* a gradient elution was used starting from 30B/70A and then changed in ten minutes to 50A/50B, with a flow rate of 1.8 mL/min. For the evaluation of* P. ginseng*, in addition to the method described above, a 90A/10B washing phase was implied for 2 minutes. The flow rate was 1.7 mL/min. For the measurements Phenomenex Kinetex C18, 150×4.6 mm, 100 Å, 5 *μ*m column, was used. In case of of* L. carthamoides* the elution started from 17.5B/82.5A isocratic flow for 1.5 minutes and then changed to 23B/77A in 6.5 minutes, followed by a washing phase with 100B/0A for 2 minutes, with a flow rate of 1.5 mL/min. The column was Phenomenex Kinetex C18, 250×4.6 mm, 100 Å, 5 *μ*m. For the quantification of* R. rosea* as stationary phase a Phenomenex Luna C18, 150×4.6 mm, 100 Å, 5 *μ*m column, was used. The solvent system consisted of 0.01% TFA (A) and acetonitrile (B), with a flow rate of 1.8 mL/min. The gradient started from 9B/91A to 17B/83A in 6 minutes, then changing to 50B/50A in 8 minutes, followed by a washing phase of 50B/50A for 2 minutes. Calibration solution series (5 concentrations each) were made from biologically active markers of the plants.

### 2.3. Viability Assay

The culturing, harvesting, and monitoring methods of* Philodina acuticornis* (PA; bdelloid rotifer) have been reported in detail in our prior publication [5]. These experiments were performed on microinvertebrates; therefore, according to the current ethical regulations, no specific ethical permission was needed. The investigations were carried out in accordance with globally accepted norms: Animals (Scientific Procedures) Act, 1986, associated guidelines, EU Directive 2010/63/EU for animal experiments, and the National Institutes of Health Guide for the Care and Use of Laboratory Animals, 1978. Our animal studies comply with the ARRIVE guidelines. In brief, the animals were cultured in standard medium (SM), a supervised and semisterile environment. Clear cultures of PA were kept in standardized cell culturing flasks, at 25°C and under a light/dark cycle of 12:12 hours. The rotifers were selected approximately 5 days after hatching (determined by body size; length 220 ± 10 *μ*m and width 60 ± 5 *μ*m), 1-2 days before the beginning of the reproductive stage. We chose 5-day-old rotifers for our measurements, when they are after the peak growth rate, at a point of inflexion of maximal size and before egg production phase [5]. After 24 h of the standard isolation process, the rotifers were treated in a 384-well plate, n=16/well/compound. For this* in vivo* experiment stock solutions of the extracts were prepared with 1% aqueous DMSO. The stock solutions were added to standard media reaching 100 *μ*M final concentrations for the compounds and 0.1% DMSO content. The untreated control group (UC) was grown in SM, while the control group (C) was kept in SM containing 0.1% DMSO (n=16, well). The status of the specimens under treatment was compared to the C group. This period lasted for 72 hours (toxicity interval), without feeding [11, 12]. The food and the feeding process are polluting factors which could intervene in the mechanism of treating agents. From the fourth day began the daily monitoring period under restricted caloric state (homogenized yeast solution, 50 *μ*g/mL) which is enough for surviving but ceases the reproduction. The viability of rotifers was assessed with three different assays utilizing video recordings with digital camera (Nikon Corp., Japan). With help of toxicity and survival lifespan assay (TSL) (n=16, well) the impact of the test compounds on the lifespan of PA rotifers was assessed. The morphological viability markers, chosen for evaluation, was defined in our previous work [5]. The body size index (BSI) (n=15, one housed rotifer) and the mastax contraction frequency (MCF, contraction/sec, 24 individual rotifers) were used as quantitative viability marker. Statistical evaluation was performed with GraphPad Prism 7.0b software, using one-way ANOVA with* post hoc *Bonferroni test. Different levels of significance were indicated as follows: p*∗∗* ≤ 0.01, p*∗∗∗* ≤ 0.001, and p*∗∗∗∗* ≤ 0.0001.

## 3. Results and Discussion

### 3.1. Characterization of the Extracts

HPLC analysis of the extracts revealed that the extract of* W. somnifera* contained 8.75±0.02 mg/g withaferin A, 0.17±0.01 mg/g withanolide A, and 0.17±0.01 mg/g withanolide B, whereas from* R. rosea* 8.26±0.13 mg/g salidroside, 1.78±0.14 mg/g tyrosol, 9.55±0.02 mg/g rosavin, and 6.28±0.05 mg/g cinnamyl-alcohol, from* P. ginseng* 5.81±0.15 mg/g ginsenosid Rb1, from.* L. carthamoides* 30.13±0.03 mg/g 20-OH-ecdysone and 15.33±0.11mg/g ajugasteron were quantified.

### 3.2. Effect on Rotifer Viability

**Figure 1:**
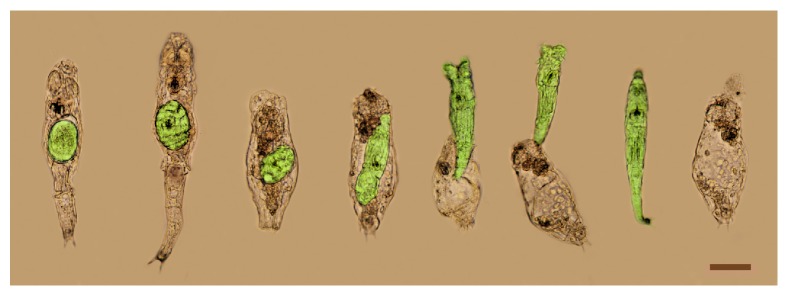
Effect of 20-OH-ecdysone on PA. The egg (green, digitally painted) growing and hatching inside the mother's body (grey). Due to the exposure of 20-OH-ecdysone, the mother was unable to lay eggs and eventually deceased; however, the egg hatched, and the young rotifer left the body. The picture was colored for the purpose of presentation. Scale bar: 50 *μ*m.

The viability of the rotifers was observed for six days after exposure to herbal extracts. The animals received no feeding for the first three days, to examine the impact of the extracts on the specimens. Previously, we observed that, in the presence of nutrient and 20-OH-ecdysone, the reproduction of the rotifers began, but they could not lay down the eggs and eventually deceased. The eggs hatched however, and the young rotifer left the mother's body (Figure 1).

**Figure 2:**
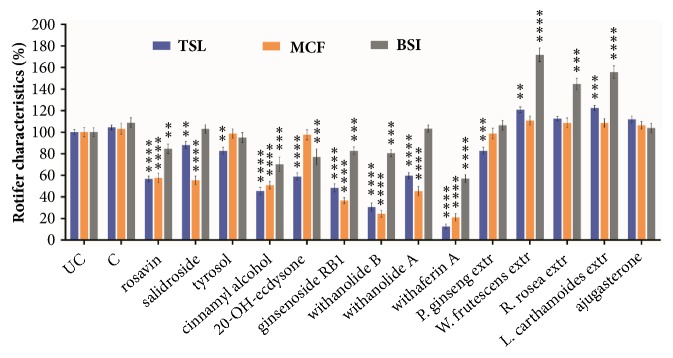
Viability values of PA treated with test substances. Changes in the viability of the PA after 6 days' treatment, compared to the C group.** UC: **untreated control.** C: **control with 0.1% DMSO.** TSL**: toxicity and survival lifespan (n=16, well).** BSI**: body size index (n=15, one housed rotifer).** MCF**: mastax contraction frequency (n=24, individual rotifer). Values are the mean ± SEM.

Three viability values were followed for a six-day period in 16 groups (Figure 2). Compounds rosavin, cinnamyl alcohol, ginsenosid Rb1, withanolide B, withanolide A, and withaferin A caused significant decrease in the number of survivors and in the MCF value. However, we observed a BSI growth amongst the survivors, probably since they utilized the compounds as nutrient. Significant increase was observed in the BSI values of groups treated with* W. frutescens* and* R. rosea* extracts, along with slight elevation in TSL and MCF. Three groups showed unique changes: exposure to compound salidroside resulted in significantly decreased MCF with normal BSI in the survivors. In the 20-OH-ecdysone group we observed 40% decrease in rotifer number with less BSI and normal MCF. From the 14 compounds, withaferin A proved to be the most toxic.

Although adaptogenic plants have been widely used for several purposes in human medicine, their exact mechanism of action and the full spectrum of active constituents have not yet been revealed. Here we publish the first results on the effects of adaptogenic plants and their constituents on viability indices of bdelloid rotifers. This well-reproducible model might be a useful tool in the characterization of bioactivities of further plants and compounds and in the identification of key components with viability-enhancing effects.

## 4. Conclusions

Whilst the crude extracts seemed to be nontoxic to PA rotifers, the pure substances of the plants were less beneficial to the rotifers and influenced negatively the viability index numbers. The aglycone tyrosol and cinnamyl alcohol were more toxic than their glycosides salidroside and rosavin. The glycoside ginsenoside Rb1 exerted deleterious effect on the animals. Although the 20-OH-ecdysone and ajugasterone C are chemically very similar, the latter was less toxic. Constituents of* W. frutescens* proved to be the most toxic compounds during the trial. Based on these results, this bdelloid rotifer model seems to be appropriate for the comprehensive testing of adaptogenic plants and their constituents.

## Data Availability

The data used to support the findings of this study are available from the corresponding author upon request.
